# The impact of heterogeneity of the air-blood barrier on control of lung extravascular water and alveolar gas exchange

**DOI:** 10.3389/fnetp.2023.1142245

**Published:** 2023-05-11

**Authors:** Giuseppe Miserocchi

**Affiliations:** Dipartimento di Medicina e Chirurgia, Università Milano-Bicocca, Monza, Italy

**Keywords:** Starling law, edema, lung diffusion, lung perfusion, lung interstitial matrix

## Abstract

The architecture of the air-blood barrier is effective in optimizing the gas exchange as long as it retains its specific feature of extreme thinness reflecting, in turn, a strict control on the extravascular water to be kept at minimum. Edemagenic conditions may perturb this equilibrium by increasing microvascular filtration; this characteristically occurs when cardiac output increases to balance the oxygen uptake with the oxygen requirement such as in exercise and hypoxia (either due to low ambient pressure or reflecting a pathological condition). In general, the lung is well equipped to counteract an increase in microvascular filtration rate. The loss of control on fluid balance is the consequence of disruption of the integrity of the macromolecular structure of lung tissue. This review, merging data from experimental approaches and evidence in humans, will explore how the heterogeneity in morphology, mechanical features and perfusion of the terminal respiratory units might impact on lung fluid balance and its control. Evidence is also provided that heterogeneities may be inborn and they could actually get worse as a consequence of a developing pathological process. Further, data are presented how in humans inter-individual heterogeneities in morphology of the terminal respiratory hinder the control of fluid balance and, in turn, hamper the efficiency of the oxygen diffusion-transport function.

## Introduction

In general, the architecture of the air-blood barrier is considered as being very effective in optimizing the gas exchange (Swan and Tawhai, 2011) by preserving the specific feature to assure it, namely its extreme thinness, that, in turn, reflects a strict control to keep lung extravascular water at minimum (Miserocchi, 2009). This occurs in physiological conditions despite the existence of morphological and mechanical heterogeneities of the terminal lung units. This review will explore how the heterogeneity in the morphology and perfusion of the terminal respiratory units come into play when the lung is facing edemagenic conditions that notably impact on the control of extravascular water and, in parallel, on the efficiency of the oxygen diffusion-transport at the level of the alveolar-capillary unit (Miserocchi et al., 2022). Further, evidence is provided concerning inter-individual differences in humans justifying the proneness to develop lung edema.

## The structure-function of the air-blood barrier

**FIGURE 1:**
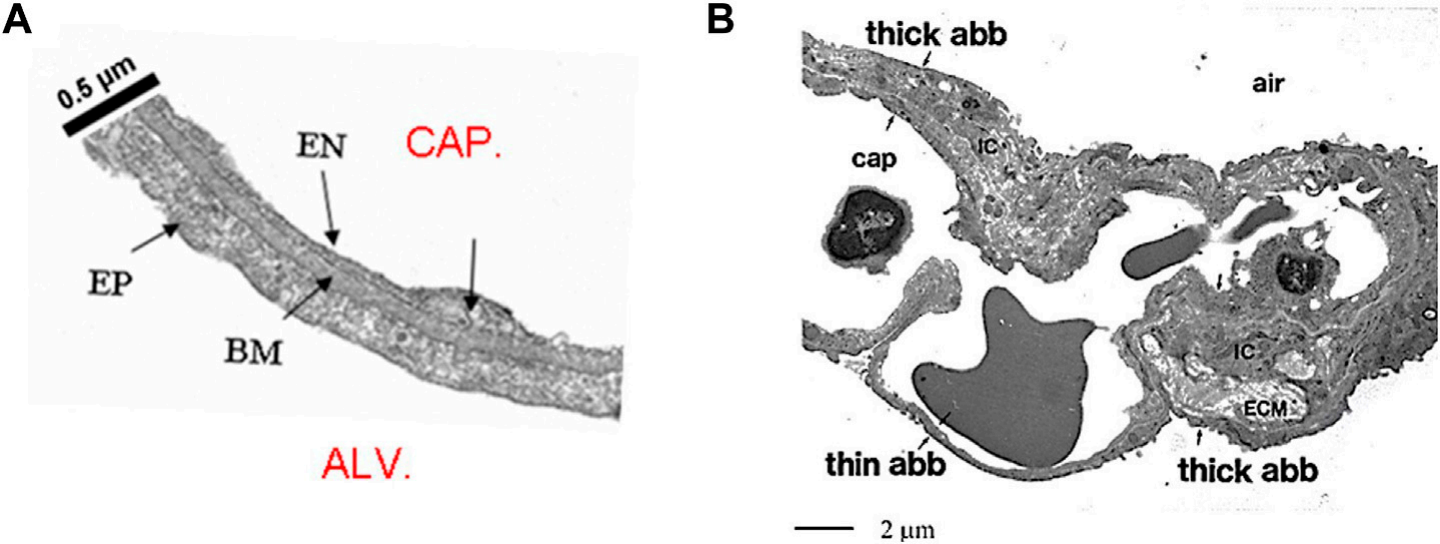
**(A)** Microphotograph of the thin portion of the air-blood barrier separating the capillary blood (CAP) from the alveolar air (ALV); endothelial cell (EN), epithelial cell (EP) and basement membrane (BM) representing the interstitial compartment (from Botto et al., 2006). **(B)** Lower magnitude TEM image to include the thick and the thin portion of the air-blood barrier (abb); IC are the interstitial cells, ECM is extracellular matrix (from Conforti et al., 2002).

The air–blood barrier represents the interface for gas exchanges; it is made of the capillary endothelium, an interstitial compartment and the alveolar epithelium. The surface area of the air-blood barrier is of the order of 2000 cm^2^/g lung tissue. Its thin portion, accounting for almost 50% of the whole surface, is <0.5 μm thick (Figure 1A) and is simply made of endothelium, epithelium and an intervening fused basement membrane representing the interstitial compartment. The overall surface of the thin portion is estimated at ∼100 m^2^ in the human lung (Weibel, 2017).

A valid representative significance of the structure-function of the air-blood barrier rests on the comparison between oxygen diffusion, that is in the range of 15 × 10^−2^ mL/(min cm^2^) at rest, while capillary microvascular filtration is at least 10,000 fold less (Miserocchi, 2009); thus, the air-blood barrier allows easy transfer of respiratory gases but it is minimally permeable to water. The diffusional oxygen uptake in the air-blood barrier ( $\left.\dot{V}O_{2}\right)$ is coupled to its transport in the blood as defined by the Fick principle:

$$\dot{V}O_{2}=\frac{Cardiac\;output}{C_{a}-C_{v}}$$

being $C_{a}\;and\;C_{v}$ the arterial and venous concentration of oxygen.

The fibrillar molecular component is mostly present in the “thick portion” of the air blood barrier (Figure 1B) and includes collagen I and III and elastic fibers (Conforti et al., 2002). The other important extracellular matrix component includes the proteoglycans (PGs). PGs consist of a “core protein” with one or more covalently attached glycosaminoglycan (GAG) chains. GAGs are linear polysaccharides made of repeating disaccharide units; they are highly polar and attract water at their hydrophilic end. GAGs are widely present in the body acting as lubricants, in particular when pressures are being generated among adjacent structures. PGs family comprises the large chondroitin sulphate sub-family (>1,000 kDa) that fills the voids between the fibrillar component. PGs act as link proteins binding with several other molecules, as well as with cell surface. The low-energy ionic and/or non-covalent bonds of chondroitin sulphate PGs with the collagen-elastin network allow reciprocal movement/sliding by reducing shear stresses during movements but also assure mechanical stability of the lung parenchyma (Cavalcante et al., 2005; Ma and Bates, 2014). In the thin portion of the basement membrane the PG-heparan sulphate sub-family (300–500 kDa) play a key role in the control on extravascular lung water assuring low microvascular permeability of the capillary surface to water and proteins as well as low porosity of the interstitial matrix (Negrini et al., 1996).

The molecular architecture of the alveolar compartment and the presence of surfactant on the alveolar surface represent stabilizing factors that redistribute forces between adjacent alveoli, so as to avoid alveolar collapse or overdistension in physiological conditions (Knudsen and Ochs, 2018).

## The Starling law governing fluid exchanges

The law governing fluid exchanges ( Jv ) between any two comparments is given by:

$$Jv=Kf∙\left[\left(P_{1}-P_{2}\right)-\sigma \left(\Pi_{1}-\Pi_{2}\right)\right]$$ (1)

being *P* and *Π* the hydraulic the colloidosmotic pressures in the compartments. Factor *σ*, named proteins reflection coefficient, is the permselectivity of the membrane to plasma proteins. The value of *σ* varies between 0 and 1, reflecting the molecular sieve of membrane pores relative to the size of the proteins. The equation includes the filtration coefficient *Kf* = *Lp∙A*, being *Lp* the hydraulic conductance and *A* the surface area available for fluid exchange. It is of interest to recall that the endothelial cells of the microvascular district, when compared to conduit vessels, exhibited a ∼20 fold lower *Lp* (Parker et al., 2006) and *σ* approaching 1 (Parker, 2007). Accordingly, concerning fluid and protein exchanges, a functional division has been proposed, between the two compartments placed in series, the “*extra-alveolar*” and the *“true alveolar*” compartment (Figure 1A), the latter being specifically designed to shield the lung against edema in order to favour gas exchange. Fluid drainage in the extra-alveolar compartment is drained by an extended lymphatic (Levick and Michel, 2010), while the “*true alveolar*” compartment, despite its incredible surface, is not served by an extended lymphatic network (Schraufnagel, et al., 2003).

Table 1 reports the estimated trans-endothelial and trans-epithelial Starling pressure gradients based on interstitial and alveolar liquid hydraulic pressure (*Pcap*, *Pi* and *Pliq alv*, respectively) and of oncotic pressure in the same compartments (*Πcap*, *Πint* and *Πliq alv*, respectively). Protein reflection coefficients for endothelium and epithelium (*σ endo* and *σ epi*, respectively) are also reported. Alveolar surface tension (γ) is in dyne/cm. In bold are hydraulic (*ΔP*) and oncotic (*σΔΠ*) pressure gradients and total Starling pressure gradient. Positive values of the Starling gradient at endothelial level indicate filtration into interstitium; negative value at epithelial level indicate alveolar reabsorption (from Beretta et al., 2021). The water content of the lung is well defined by the wet-to-dry weight ratio (*W/D,* index of amount of extravascular water) that, in physiological conditions, is ∼5 (Miserocchi et al., 1993). The thinness of the air-blood barrier reflects a strict control of extravascular water volume that is maintained at minimum, thanks to the extremely low water permeability (*Lp*) and a relatively high value of *σ* of the endothelial and the epithelial barriers.

**TABLE 1:** Trans-endothelial and trans-epithelial Starling pressure gradients at end-expiration (FRC) in physiological condition.

	Trans-endothelial		Trans-epithelial
	End-expiration		End-expiration
*Pcap*	9	*Pint*	−10
*Pi*	−10	*Pliq alv*	∼0
*σ endo*	0.85	*σ epi*	0.85
*Πcap*	26.8	*Πint*	13.8
*Πint*	13.8	*Πliq alv*	0
		γ	1
***ΔP***	**19**	***ΔP***	**−10**
***σ·ΔΠ***	**−11.0**	***σ·ΔΠ***	**−11.7**
**Starling gradient**	**8.0**	**Starling gradient**	**−21.7**

Pressure values are expressed in cmH_2_O; σ is a pure number. Surface tension γ = 1 dyne/cm. In bold, hydraulic (ΔP) and oncotic (σΠP) pressure gradients and total Starling pressure gradient. From (Beretta et al., 2021).

**FIGURE 2:**
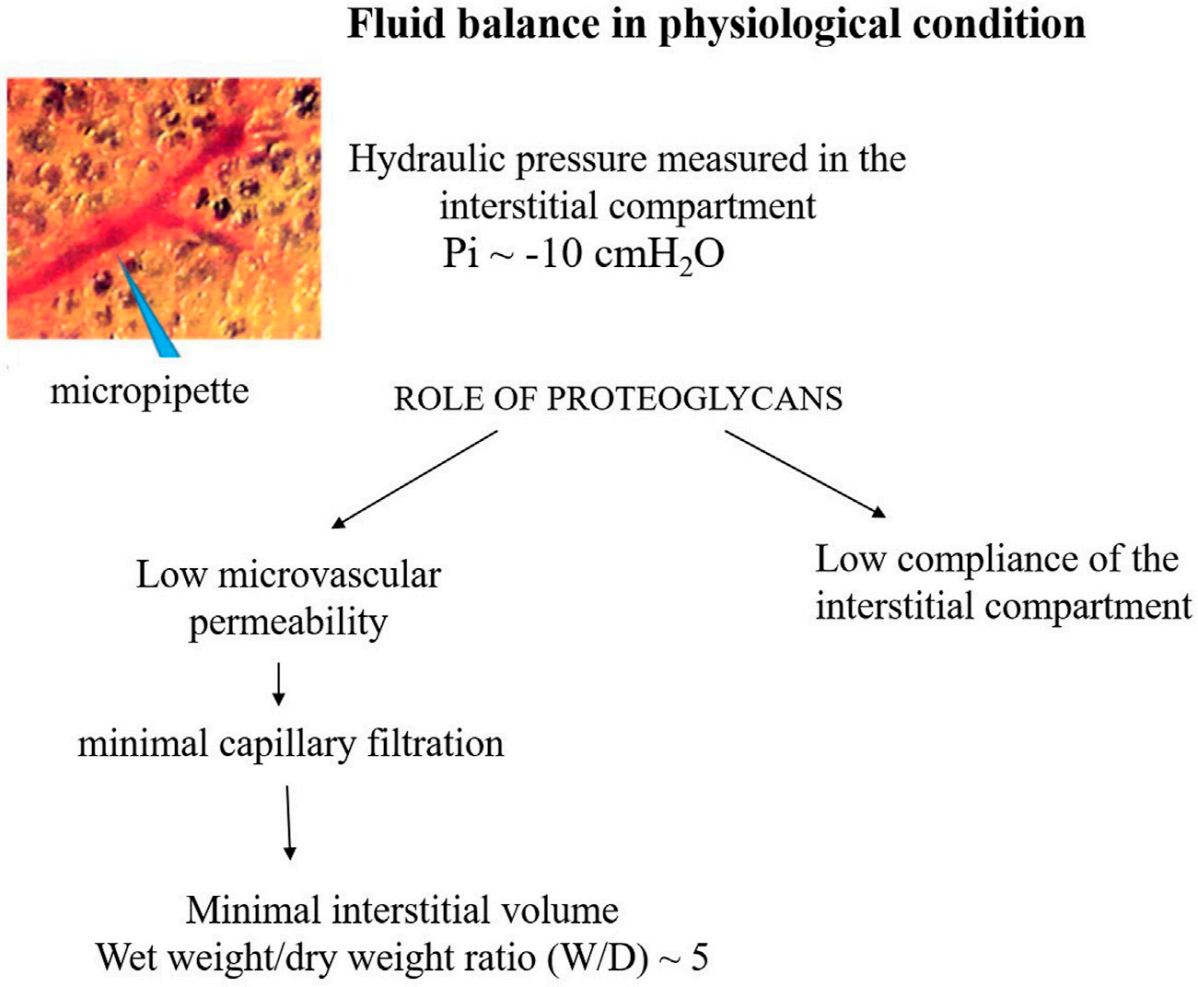
Conceptual diagram depicting the fluid dynamic and mechanical features involved in the control of lung fluid balance in physiological conditions.

Figure 2 presents a conceptual diagram to depict the fluid dynamic and mechanical features involved in the control of lung fluid balance in physiological conditions. The interstitial compliance, defined as the ratio of the change in interstitial volume to the corresponding change in *Pi*, is also indicated; its low value in physiological conditions, 0.2 mL mmHg^-1^ 100 g^-1^, hinders the accumulation of interstitial fluid (Miserocchi et al., 2001).

### The intrinsic resistance of the lung to developing edema

Experimental models of edema were developed by slowly inducing a perturbation in fluid balance, this allowed to study the time course of changes in mechanical structure/properties of the interstitial compartment to affect the control on the volume of extravascular water. These kind of informations could not be provided by experimental approaches causing acute severe lesion.

**FIGURE 3:**
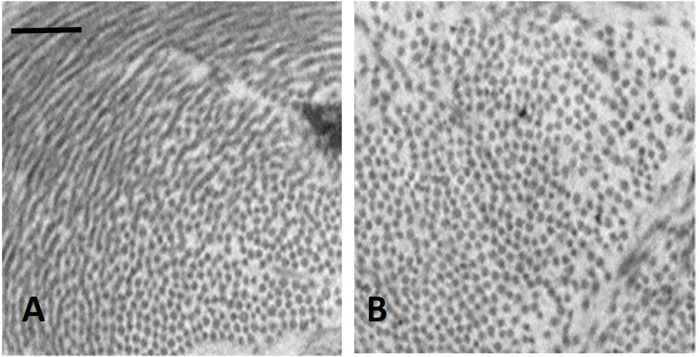
TEM image of clusters of cross and longitudinally sectioned collagen fibrils in the interstitium of the alveolar septa in the less dependent region of a control lung **(A)** and in the more dependent region after inducing *interstitial edema* **(B)** Scale bar 450 nm (from Conforti et al., 2002).

It was found that in the initial phase of developing lung edema, water filtering in the interstitium is captured by the hyaluronan-versican complex to form gel, whose increase in steric hindrance raises the hydraulic interstitial pressure from −10 cmH_2_O up to ∼ +5 cmH_2_O, reflecting the low tissue compliance (Miserocchi et al., 1993; Negrini et al., 1996; Miserocchi et al., 2001). The corresponding increase in extravascular lung water, defined as *interstitial edema*, averaged ∼10%, from 5 to 5.5 *W/D*. On fluid dynamic basis, the increase in interstitial pressure due to low lung tissue compliance counters filtration, representing therefore a strong “*safety factor*” (Figures 3A, B). The condition of *interstitial edema* is likely quite effective, providing a satisfactory answer to the question posed by Hopkins (2010): “*given the sporadic nature of exercise-induced edema … why do not more athletes develop alveolar flooding during exercise?*”

A major question remains: how long can the lung resist in sustained edemagenic conditions?

The kern of severe edema formation is the progressive loss of function of PGs families causing the waning of the “*safety factor*”.

Figure 3 shows clusters of cross sectioned collagen fibrils in the interstitium of the alveolar septa in the less dependent region of a control lung (Figure 3A) and in the more dependent region after inducing interstitial edema (Figure 3B) (data from Conforti et al., 2002). The white voids surrounding the fibrillary component are occupied by PG-chondroitin sulphate sub-family. Clearly, interstitial edema caused an increase in inter-fibrillar distance of variable extent, roughly doubling from ∼50 to ∼100 nm (Conforti et al., 2002). Intermolecular forces are governed by the inverse-square law so that attraction force falls off with the square of the distance between the interacting molecules. Accordingly, were the estimates relative to interstitial hydration representative of the average increase in intermolecular distance, the force of the non-covalent bond would fall to 1/4 of its original strength.

**FIGURE 4:**
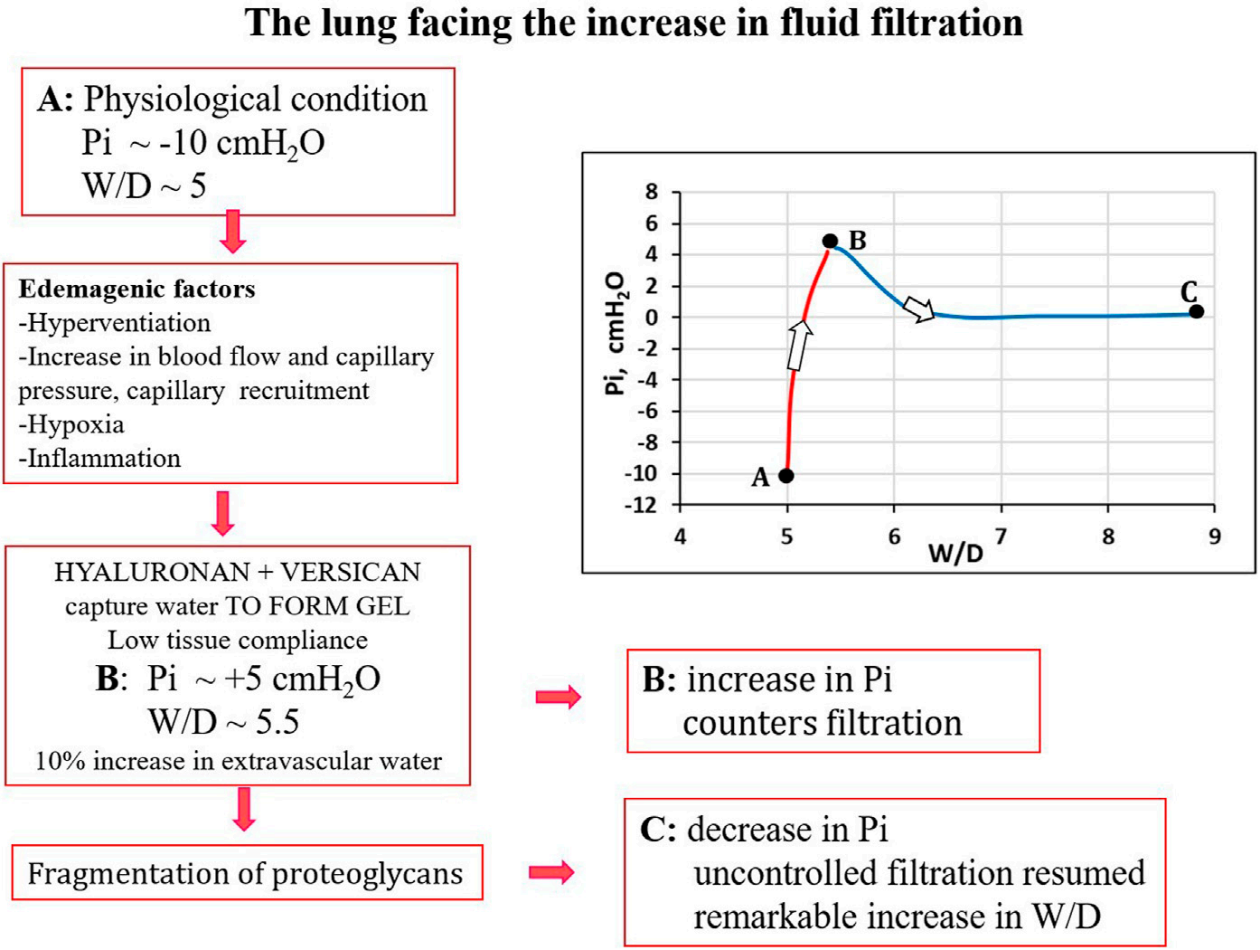
Conceptual diagram showing pulmonary interstitial pressure (*Pi*) as a function of *W/D*. Control conditions **(A)**, *interstitial edema* **(B)**; the role of fragmentation of proteoglycans is highlighted in the development of severe edema **(C)**.

Besides loosing force of bonds, fragmentation of PGs also occurs down a sequence of events including the production of reactive oxygen species (ROS) due to inflammation (bacterial, viral or of sterile type, e.g. hypoxia, surgery) and the activation of metallo-proteases (Negrini et al., 1996; Negrini et al., 1998; Miserocchi et al., 1999; Passi et al., 1999). Fragmentation of intermediate/small PGs leads to progressive increase in microvascular permeability to water (increase in *Lp*) and solutes (decrease in *σ*). Figure 4 summarizes the events leading to the development of severe lung edema; in particular, fragmentation of large PGs vanish the increase in interstitial pressure (increase in tissue compliance) giving rise to uncontrolled filtration (Figures 4B, C).

**FIGURE 5:**
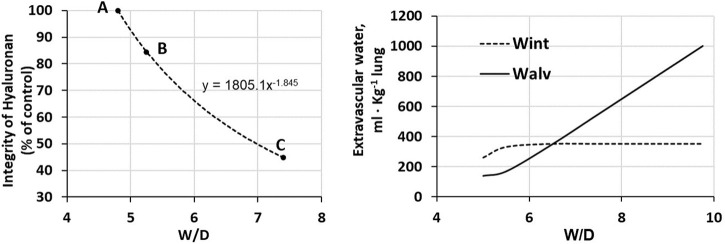
LEFT. Relationship between loss of integrity of interstitial hyaluronan and increase in *W/D* ratio of the lung. Points A, B and C refer to the conditions presented in Figure 4. RIGHT. Amount of extravascular lung water in the interstitial (Wint) and alveolar (Walv) compartments on increasing *W/D* (data from Beretta et al., 2021).

Total proteoglycan content of the lung was evaluated by hexuronate assay on tissue samples. The technique to estimate the integrity of PGs was based on their extractability from the interstitial matrix using a chemical agent (guanidinium hydrochloride). It was found that proteoglycan extractability increased with increasing time using different models of edemagenic exposure, suggesting a weakening of noncovalent bonds linking intact proteoglycans to other interstitial matrix components (Negrini et al., 1996; Negrini et al., 1998; Passi et al., 1999; Miserocchi et al., 2001). Data allowed to derive the decrease of integrity of PGs, relative to a control value in physiological conditions (118 ± 4 μg/g wet tissue). Figure 5 LEFT shows that the loss of integrity of PGs correlates with increase in *W/D* ratio (points A, B and C refer to the conditions presented in Figure 4). Molecular size distribution of extracted PGs was also determined revealing a progressive decrease of large towards smaller molecular size suggesting fragmentation of native molecules (Negrini et al., 1998).

Figure 5 (RIGHT) shows the distribution of water in the interstitial (*Wint*) and in the alveolar compartment (*Walv*) with increasing *W/D*. Note that at *W/D* of ∼6.5, the capacity of the interstitial compartment for fluid accumulation (*Wint*) is saturated while alveolar flooding (*Walv*) will continue to increase on increasing *W/D*. Accordingly, a *W/D* of ∼6.5, corresponding to a loss of integrity of hyaluronan from 100 down to ∼55%, represents a potential threshold for alveolar flooding and thus development of severe edema. Exposure to 3–6 h of strongly edemagenic conditions impact remarkably on the integrity of the macromolecular PGs structure. Accordingly, identifying valid markers of developing edema appears crucial in the clinical setting as the time constant for developing of the severe stage ranges 4–7 min (Parker and Townsely, 2004; Mazzuca et al., 2016).

Deactivation of surfactant is a further cause leading to lung edema; this occurs on cyclic recruitment/de-recruitment of collapsed and/or fluid-filled alveoli that generate inter-alveolar forces that favour epithelial lesion and increase in microvascular permeability (Hamlington et al., 2018).

### Heterogeneity of alveolar morphology, mechanical compliance and perfusion

We will now explore how the heterogeneity of the terminal respiratory units come into play when the lung is facing edemagenic conditions.

Heterogeneity in alveolar size has been reported in the healthy experimental animal based on direct imaging of subpleural alveoli and by optical coherence tomography (Meissner et al., 2010).

**FIGURE 6:**
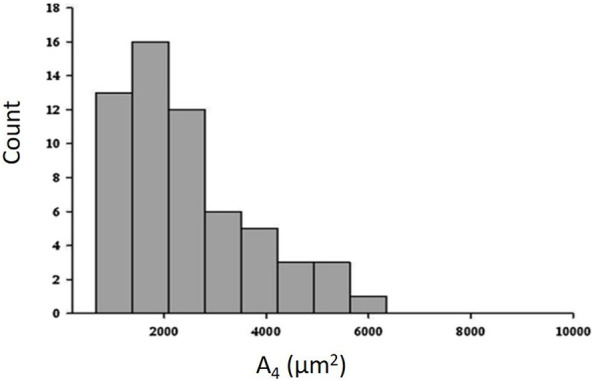
Frequency distribution of cross-sectional area (A_4_) at a transpulmonary pressure of 4 cm H_2_O (data from experimental animal; from Salito et al., 2014; Mazzuca et al., 2014).

Figure 6 shows that the distribution of cross sectional area (data taken at equal height relative to the bottom of the lung) is remarkably right skewed at a distending pressure of 4 cmH_2_O; smaller alveoli (<2000 μm^2^) are ∼6 times more abundant than larger ones >4,000 μm^2^) (data from the experimental animal, Salito et al., 2014; Mazzuca et al., 2014).

Heterogeneity in alveolar morphology implied heterogeneity in alveolar compliance in the range of transpulmonary pressure from 4 up to 8 cmH_2_O (Mazzuca et al., 2014; Salito et al., 2014).

**FIGURE 7:**
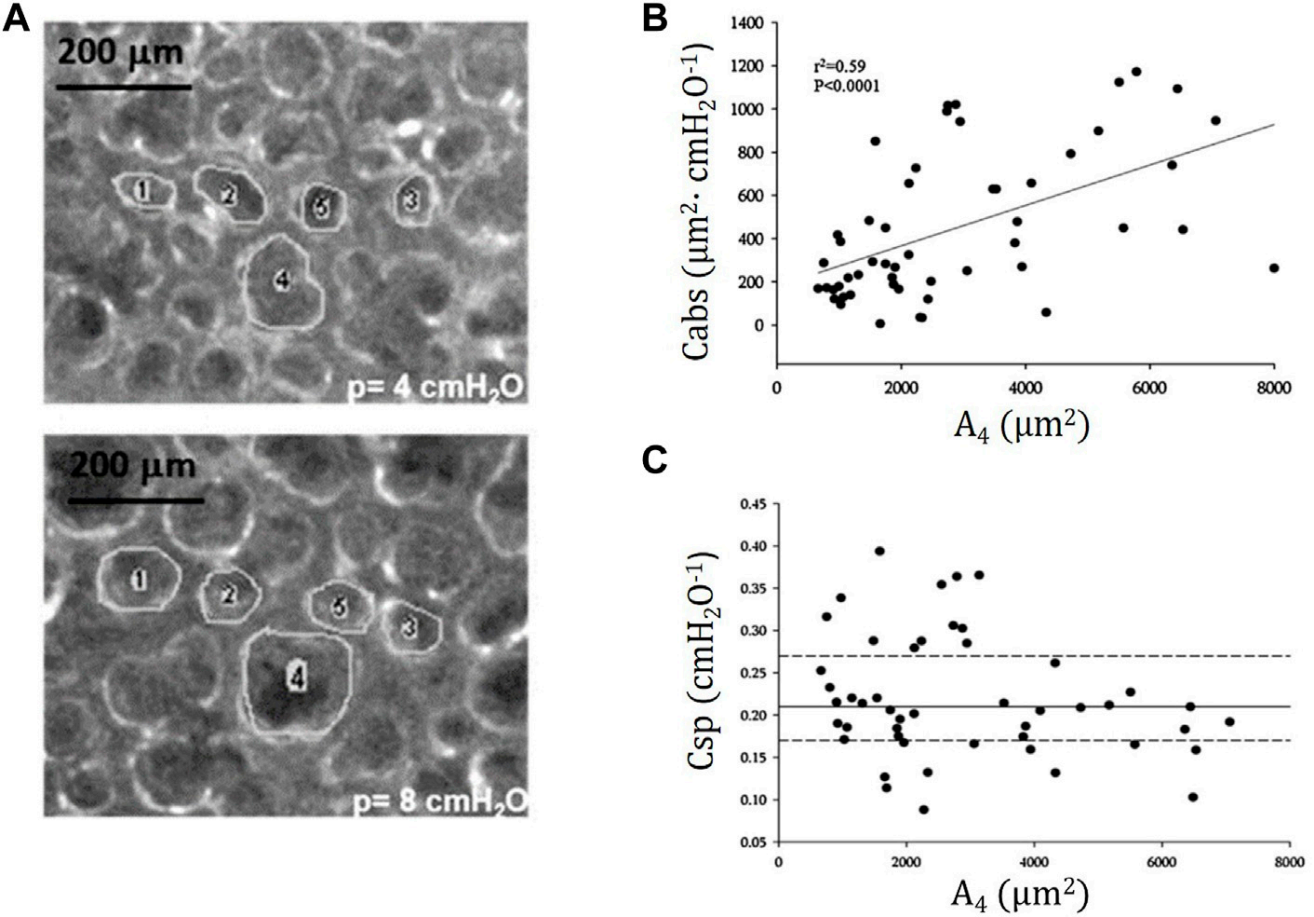
**(A)** Trans-pleural images of alveoli of different cross-sectional area at distending pressure of 4 and 8 cmH_2_O. **(B)** Absolute alveolar compliance *Cabs* (ratio of change in alveolar surface to the change in distending pressure, $\frac{∆A}{∆P})$ plotted as a function of alveolar surface area at distending pressure of 4 cmH_2_O (A_4_); **(C)** Specific alveolar compliance *Csp* ( $\frac{∆A}{∆P}∙\frac{1}{A4}$ ) plotted vs*.* A_4_ (data from Salito et al., 2014).

Figure 7A shows trans-pleural images of alveoli of different size at distending pressure of 4 and 8 cmH_2_O. Figure 7B shows that, despite some variability, an index of absolute alveolar compliance ( $\frac{∆A}{∆P})$ increases on increasing distending pressure when plotted vs*.* cross sectional area at 4 cmH_2_O (A_4_). Data from Figure 7C suggest that specific alveolar compliance (ratio of absolute compliance to A_4_, $\frac{∆A}{∆P}∙\frac{1}{A4}$ ) tends to a constant value, despite some considerable variation. The heterogeneity of alveolar size and of corresponding mechanical behaviour were fairly homogenously distributed, scaling up from small (10–20 alveoli) to larger cluster of alveoli (30–40 alveoli) (Mazzuca et al., 2014). It was suggested that the intertwining of the clusters would result in a substantially homogenous lung mechanical behaviour. The relative constancy of specific compliance might actually contribute to reduce inter-regional differences in parenchymal and surface forces by assuring a fairly uniform stretching in a model of mechanically inter-dependent alveoli (Salito et al., 2014). Yet, a further study demonstrated the existence of heterogeneity of alveolar size by administering exogenous surfactant in physiological non-surfactant deprived conditions (Salito et al., 2015). Indeed, surfactant instillation increased up to ∼50% the surface area of alveoli smaller than 2000 μm^2^ (reflecting a lowering of surface tension due to local surfactant enrichment, while, conversely, the surface area of adjacent alveoli greater than 2000 μm^2^, decreased by ∼5%. Opposite changes in alveolar surface suggest a decrease in the tethering effect exerted by the smaller alveoli on the larger ones. Exogenous surfactant delivery proved indeed to reduce alveolar stress heterogeneity (Knudsen and Ochs, 2018). Noteworthy, recent finding by Lettau et al., 2022) provide proof for heterogeneous surfactant organization of tubular myelin networks showing variously curved membrane planes with reciprocal intersections. Heterogeneity also has been documented for alveolar epithelial Type II as different subpopulation have been identified concerning their role in repair depending on the type of lesion (Lynch et al., 2019; Chen and Liu, 2020).

Interestingly, in lesional lung models causing severe edema (bleomycin treatment or surfactant deactivation by Tween 20), remarkable heterogeneity was observed ranging from atelectasis to over-distention of adjacent alveoli. This caused alveolar instability due to increased forces *via* mechanical interdependence exacerbating the spreading of lung edema during cycling recruitment/derecruitment of the alveoli (Bates & Smith, 2018; Bates et al., 2020; Gaver et al., 2020). Also in emphysema, a pathology implying high risk of edema, the heterogeneity in alveolar size, a specific trait of the disease, was greatly enhanced (Mishima et al., 1999). Further, a model simulation of heterogeneous distribution of molecular fragmentation leading to emphysema, correlated with the development of remarkable heterogeneity in alveolar size (Suki et al., 2003).

Concerning lung perfusion, extensive heterogeneities are reported in physiological conditions. Analysis of results in the upright posture, with and without gravity, and in the inverted, prone, and supine postures reveal significant flow heterogeneity and a persistent decrease in flow in the cranial and caudal regions for all postures suggesting that vascular geometry makes a major contribution to regional flow with gravity having a lesser role. A computational model of blood flow through the human pulmonary arterial tree suggested that, besides gravity and posture, the geometry of the branching structure plays a significant role in flow heterogeneity (Burrowes and Tawhai, 2006). Further, fractal geometry analysis showed that small asymmetries along the branching system might account for heterogeneity in both airways flow and blood flow distribution in terminal units (Glenny and Robertson, 2011). The last paper also confirmed that the regional lung perfusion reflects the complex interaction between vascular and surrounding alveolar pressures, particularly when local vasoconstriction occurs in response to hypoxia.

In *interstitial edema,* the immediate response is precapillary vasoconstriction, involving arterioles ∼80 µm in diameter (Negrini et al., 2001). The functional significance of this reflex is to avoid an increase of capillary pressure relative to the physiological value (∼10 cmH_2_O), particularly when capillary recruitment occurs on cardiac output as a response to increased oxygen requirement. A comprehensive model of fluid exchange at capillary level (based on Eq. 1, suggests indeed that doubling capillary pressure (say from 10 up to 20 cmH_2_O) is, by and large, more edemagenic compared to an eight fold increase in water permeability *Lp* (Mazzuca et al., 2016). The reason is that the increase in pressure contributes to capillary recruitment, thus implying also an increase in *Kf* due to increase *A* (see Eq. 1).

**FIGURE 8:**
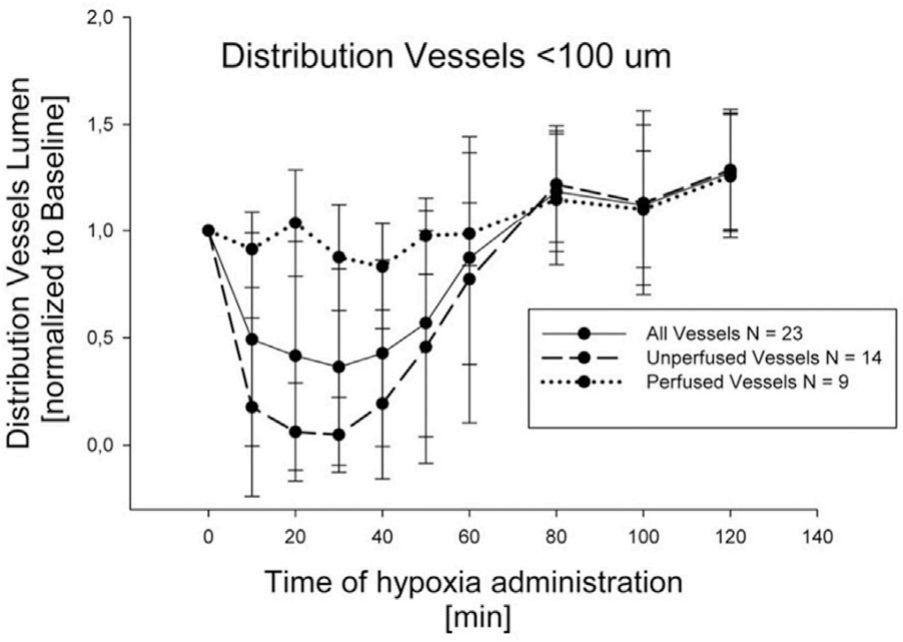
Time course of vasomotion in arterial pulmonary distribution vessels with diameter <100 nm in response to hypoxia (12% oxygen) (from Mazzuca et al., 2019).

The lung vasomotion in distribution vessels <100 µm in response to a strong edemagenic factor (hypoxia) is shown in Figure 8, actually revealing two different patterns: either no change, or remarkable decrease in diameter approaching complete closure after 30 min of hypoxia exposure (Mazzuca et al., 2019). Closure was followed by return towards control at about 80 min, remaining thereafter essentially steady. On average, considering all the data, a significant decrease in diameter down to about 50% of control was observed at 30 min.

Greater vasoconstriction occurs in regions developing edema with redirection of blood flow from edematous to non edematous regions (Rivolta et al., 2011; Mazzuca et al., 2019).

An attempt was also done to model the alveolar capillary network after identifying corner vessels and assuming a random distribution of Voronoi points (Mazzuca et al., 2019); the number of points was chosen so as to match the ratio between capillary volume and interstitial volume. The functional significance of this ratio is that to balance the filtration surface of the capillary network with the capacity of the surrounding interstitial compartment to host microvascular filtration. An estimate of this ratio, based on TEM images (Conforti et al., 2002), was set at 1.54.

**FIGURE 9:**
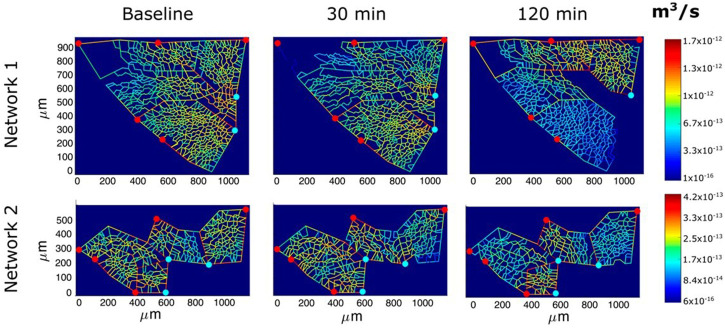
Perfusion analysis for alveolar-capillary units relative to two networks (1 and 2). Red and light blue dots identify respectively arteriolar accesses and venular exits. The color panels show the capillary blood flow at different time points (baseline, 30 min and 120 min of hypoxia exposure) with color-coded log-scale intensity (from Mazzuca et al., 2019).

Figure 9 shows an example of the capillary perfusion pattern with a color-coded log-scale in alveolar districts after inducing hypoxic edema (Mazzuca et al., 2019). Data refer to two different networks indicated as 1 and 2. Progressive closure of microvessels (blue colour) was observed over time in alveolar regions where interstitial edema was more marked, as judged from the thickness of the perivascular interstitial space. Blood flow switching was described as reflecting the geometry of branching, as well as the transcapillary pressure (capillary pressure—interstitial pressure) along the longitudinal profile of the capillary. The model supports the heterogeneous distribution of edema further suggesting that capillary closure is favoured by the compressive action of the increase in interstitial pressure, as predicted by the model of Glenny and Robertson, 2011). The heterogeneity in edema development was found to correlate with uneven pulmonary blood flow distribution (Urano et al., 2005). Capillary de-recruitment due to perivascular edema may well occur also in humans, as administration of a vasodilator agent was unable to restore blood flow in edematous lung regions (Scherrer et al., 1996).

A recent study (Buxton et al., 2022) provides data from a novel analysis on the spatial/temporal dynamics of blood flow in the human lung measured noninvasively, potentially useful to estimate the vascular control mechanisms involved.

Finally, semi-quantitative estimates of microvascular filtration were obtained from a theoretical model of the morphologically based–alveolar capillary unit (Mazzuca et al., 2016). Giving the heterogeneous distribution of blood flow, it was found that the filtration flow was also heterogeneously distributed. A key point of this analysis was that greater microvascular filtration occurred around alveoli of larger size. Although TEM images were not available, one may hypothesize that, on geometrical ground, the capillary surface available for filtration in large alveoli is actually oversized compared to the capacity of the interstitial compartment to host microvascular filtration.

### Inter-individual difference in human alveolar-capillary phenotype as potential factor to develop lung edema

As long as the architecture of the air-blood barrier is preserved, it appears to be very effective in optimizing the gas exchange (Swan and Tawhai, 2011). It is a common experience however that, in edemagenic conditions, some subjects are more prone to develop lung edema compared to others. The characteristic example is the exposure to hypoxia at high altitude that allowed to distinguish between HAPE-S (High Altitude Pulmonary Edema-Sensitive) and HAPE-R (High Altitude Pulmonary Edema-Resistant) subjects (Busch et al., 2001; Dehnert et al., 2006; Richalet et al., 2012; 2021; Eichstaedt et al., 2020a; b). Further, studies based on the MIGET technique (Multiple Inert Gas Elimination Technique, Wagner, 2008) have reported remarkable inter-individual differences in the perturbation of the distribution of the VA/Q ratio that were attributed to regional development of lung edema in severe edemagenic conditions (exercise in hypoxia) (Hammond et al., 1986; Hopkins et al., 1996; Hopkins, 2005; Hopkins, 2020).

**FIGURE 10:**
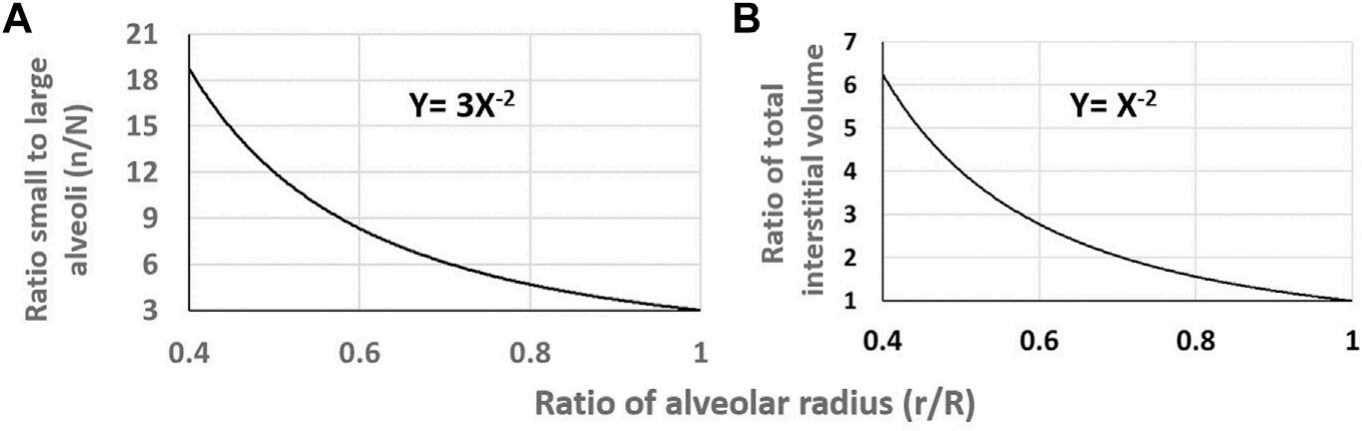
**(A)** Relative increase in the ratio of number of alveoli on decreasing $\frac{r}{R}$ from 1 to 0.4.**(B)** Relative increase of the peri-alveolar interstitial compartment volume on decreasing $\frac{r}{R}$ from 1 to 0.4.

Since lung edema is primarily affecting the alveolar-capillary compartment, a line of research aimed at characterizing inter-individual differences concerning the oxygen diffusion/transport function. At morpho-functional level, subject were found to differ remarkably considering the ratio between the pulmonary capillary blood volume (*Vc*) and the alveolar membrane diffusive capacitance (*Dm*) (Miserocchi et al., 2008). At the functional respiratory capacity (FRC, corresponding to end-expiration), the *Vc/Dm* ratio was found to vary from ∼ 1 up to ∼ 6, with a normal distribution (Miserocchi et al., 2022). It was considered that subjects with low *Vc/Dm* have a less extended capillary network relative to the surface of the air-blood barrier; on the opposite, subjects with high *Vc/Dm* have a more extended capillary network compared to the surface of the air-blood barrier. At FRC people with low *Vc/Dm* ratio, had, on the average, a 3 times greater *Dm* value (Miserocchi et al., 2008) likely reflecting a greater alveolar surface. It was considered that a higher value of *Dm* would be justified by a greater number of alveoli with smaller radius (set as *n* and *r*, respectively), compared to the case of low *Dm* implying alveoli of smaller number but greater radius (*N* and R, respectively). For a ratio between high and low *Dm* set at 3, and assuming either a spherical shape or regular polyhedron for the alveoli, the ratio of alveolar surfaces can be numerically modelled, being proportional to the product $\frac{r^{2}}{R^{2}}\frac{n}{N}=3.$ Figure 10A shows that by decreasing the ratio $\frac{r}{R}$ from 1 down to 0.4, the number of alveoli would increase as a power function. Assuming now a constant thickness of 0.1 µm for the peri-alveolar interstitial compartment, one can estimate the relative increase in volume of the interstitial compartment on decreasing $\frac{r}{R}$ . Figure 10B shows that for $\frac{r}{R}=0.4$ , the overall perialveolar interstitial volume would increase ∼6 fold.

One can therefore hypothesize that a greater capacity of the peri-alveolar interstitial volume to accept fluid in edemagenic condition would help to prevent alveolar edema; this would be the case for a greater number of alveoli of smaller radius. This concept fits with the experimental finding of greater interstitial fluid accumulation around large alveoli (Mazzuca et al., 2019).

The potential advantage of small alveoli in favouring gas exchange has been considered based on a geometrical approach similar to that proposed for the interpretation of differences in *Dm*. A critical parameter optimizing gas-exchange would be the perimeter of the surface on a planar cut of the acinus; the conclusion by the authors is that “*the best possible acinus*” should be small so that the length of its perimeter is of the order of the length travelled by a gas molecule before being absorbed (Sapoval et al., 2002; Felici et al., 2003).

Considering oxygen uptake, model by Swan and Tawhai, 2011, suggested that, despite some morphological heterogeneity, it is very efficient at rest, with only a small degree of intra-acinar PO_2_ heterogeneity. Further, it was recognized that anisotropy due to deformation causing non-uniformity of the tissue stretch among alveoli, could become important to hinder oxygen uptake only at high rates of ventilation (Swan et al., 2012).

### Inter-individual heterogeneity of the air-blood barrier in humans and its impact on oxygen uptake-transport in edemagenic conditions

It is easy to hypothesize that edema developing at the level of the air-blood barrier might affect the efficiency of oxygen diffusion/transport. Given the differences in human alveolo-capillary phenotype, as suggested by the *Vc/Dm* ratio (Miserocchi et al., 2008), and the potential correlation with the control of lung fluid balance, a line of research was dedicated to identify inter-individual differences in the response to edemagenic conditions. The study relied on a biophysical model defining the alveolar-capillary oxygen equilibration (*Leq*) in the air-blood barrier (Piiper and Scheid, 1981) as:

$$Leq\;{=e}^{-\frac{{DO}_{2}}{\beta \dot{Q}}}$$ (2)

being *DO*_2_ the O_2_ diffusive capacity, $\dot{Q}$ cardiac output and *β* is the Hb binding capacity for O_2_.

*Leq* defines the equilibrium reached in the blood exiting the pulmonary capillaries between the PO_2_ in the arterial blood relative to the alveolar PO_2_. *Leq* varies from 0 (the case where PO_2_ in the arterial blood attains alveolar PO_2_) to 1 (the case of arterial-venous shunt).

The model assumes a kinetics of equilibration of exponential type, accordingly Eq. (2) can be rewritten as:

$$L_{eq}=e^{-\frac{Tt}{\tau }}$$ (3)

being *Tt* the blood transit time in the pulmonary capillaries that can be estimated as $Tt=\frac{Vc}{\dot{Q}}$ , being *Vc* the pulmonary capillary volume and $\dot{Q}$ the cardiac output. By substitution into the exponent $\frac{{DO}_{2}}{\beta \dot{Q}}$ , one can derive $\tau =\frac{\beta Vc}{{DO}_{2}}$ .

**FIGURE 11:**
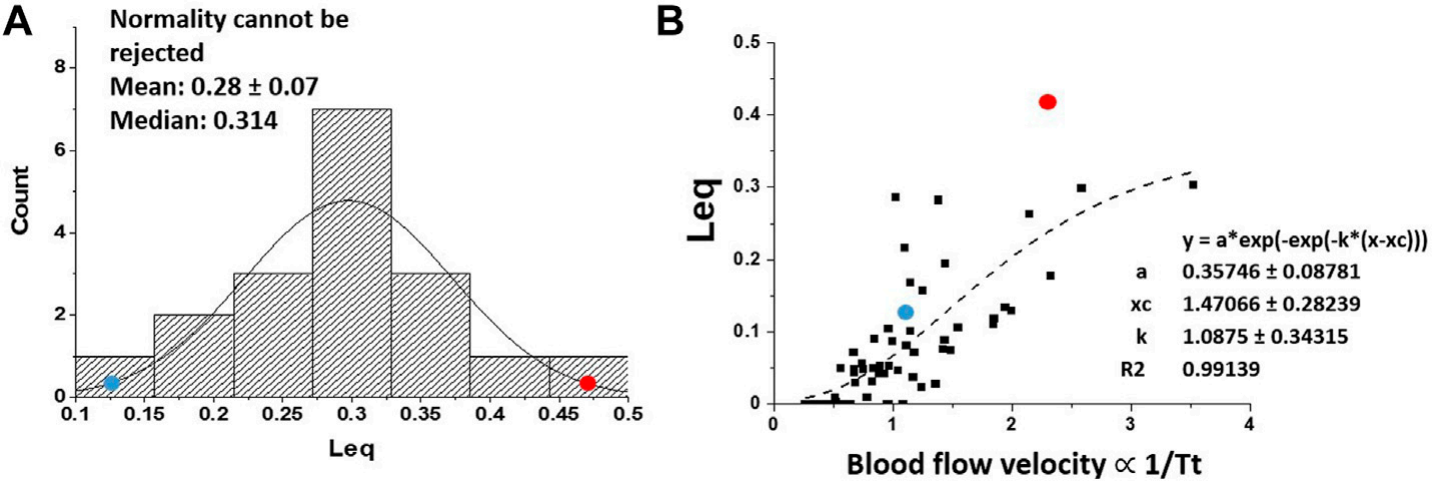
**(A)** Distribution of *Leq* among subjects exposed to severe edemagenic conditions (work in hypoxia). The blue point at the left refers to a subject with *Vc/Dm ∼* 1 at FRC at rest attaining an optimal equilibration for O_2_ exchange (with no perturbation in lung water), while the red point to right refers to a subjects with *Vc/Dm ∼* 4.3 showing that equilibration was only achieved at ∼45%. **(B)** Plot of *Leq* vs. *1/Tt* (an index of blood velocity). Red and blue dots refer to the same subjects as in **(A)** (from: Miserocchi et al., 2022).

All variables appearing in Eqs. 2, 3 could be estimated allowing to describe the kinetics of alveolar-capillary equilibration. This approach, reflecting the combined changes of the variables involved, allowed to characterize at individual level the efficiency of the oxygen diffusion-transport in edemagenic condition. The analysis was carried out in subjects at rest and exposed to strongly edemagenic conditions (work at 3,840 m, P_I_O_2_ ∼90 mmHg) (Beretta et al., 2017; Beretta et al., 2019). Subjects with low *Vc/Dm* at rest still attained *Leq* ∼ 0. Conversely, subjects having a high *Vc/Dm* showed a remarkable pulmonary capillary de-recruitment (decrease in *Vc*) and a decrease in *DO*_*2*_. The latter could be attributed to a perturbation in lung fluid balance that was assessed by the changes in mechanical properties of the respiratory system estimated with the frequency oscillation technique (Dellacà et al., 2008; Bartesaghi et al., 2014). Figure 11A shows that the distribution of *Leq* among subjects in severe edemagenic conditions is normal. The blue point at the left refers to a subject with *Vc/Dm ∼* 1 at FRC at rest attaining an optimal equilibration for O_2_ exchange (no perturbation in lung water). The red point to the right refers to a subjects with *Vc/Dm ∼* 4.3 showing that equilibration only achieved 45%. Due to impairment of the gas exchange, subjects with high *Vc/Dm* were forced to increase cardiac output. This reflex response, adding to the de-recruitment of the capillary network, led to an increase in blood velocity and shortening of Tt that prematurely truncated the equilibration process causing *Leq* to shift towards 1.

Figure 11B supports the functional correlation between the increase in *Leq* and an index of increase in capillary blood velocity expressed by the reciprocal of transit time *1/Tt*.

In general, one shall note that τ tends to be stable on varying conditions as the changes in *Vc* and *DO*_*2*_ are coherent: they either both increase (subjects with low *Vc/Dm*) or decrease (subjects with high *Vc/Dm*). Accordingly, *Tt* appears to be the more sensitive factor affecting the equilibration process (a case defined as shunt-like effect).

Besides alveolar morphological heterogeneity one shall mention a further pathophysiological factor to possibly explain a greater proneness to develop lung edema, namely an inborn greater microvascular permeability. A basic, so far unanswered pathophysiological question, remains indeed at clinical level: why does pulmonary hypertension develop in intrinsic lung disease such as idiopatic pulmonary fibrosis, interstitial pulmonary fibrosis associated with pulmonary artery fibrosis, combined pulmonary fibrosis and emphysema (Munson, 2010)? A possible answer would come by hypothesizing a common pathophysiological interpretation for these conditions. One may comment that precapillary vasoconstriction, as a reflex protective response against lung edema, inevitably leads to pulmonary hypertension. One could hypothesize that the deposition of fibrotic tissue could also be regarded as a long-term functional adaptive response to protect against the risk of lung edema in case of high inborn microvascular permeability. In support of this interpretation is the finding that the relative ratio of capillary area to total area of alveolar walls (the case of high *Vc/Dm*) was significantly higher at low grades of fibrosis than in control healthy lungs (Ebina et al., 2004). The same study reports that vascular density gradually decreased with increasing the degree of fibrosis becoming definitely lower than that of control in the most extensive lung fibrotic lesions. Obviously, if decreasing capillary surface helps preventing the occurrence of lung edema, on the other side, the complication is that excessive deposition of fibrotic tissue along the arterial vascular segment occurs at the expense of increasing right-ventricular afterload (a maladaptive case of compensation). The transdifferentiation of lung fibroblasts into myofibroblasts proliferation and extensive deposition of extracellular matrix are currently the object of growing interest from a therapeutic point of view in the pathogenesis of pulmonary fibrosis (Gan et al., 2022; Li et al., 2022; Gu et al., 2023; Qi et al., 2022).
